# Climate change impacts on the energy system: a review of trends and gaps

**DOI:** 10.1007/s10584-018-2265-4

**Published:** 2018-08-06

**Authors:** Jennifer Cronin, Gabrial Anandarajah, Olivier Dessens

**Affiliations:** Energy Institute, University College London, London, WC1H 0NN UK

## Abstract

Major transformation of the global energy system is required for climate change mitigation. However, energy demand patterns and supply systems are themselves subject to climate change impacts. These impacts will variously help and hinder mitigation and adaptation efforts, so it is vital they are well understood and incorporated into models used to study energy system decarbonisation pathways. To assess the current state of understanding of this topic and identify research priorities, this paper critically reviews the literature on the impacts of climate change on the energy supply system, summarising the regional coverage of studies, trends in their results and sources of disagreement. We then examine the ways in which these impacts have been represented in integrated assessment models of the electricity or energy system.

Studies tend to agree broadly on impacts for wind, solar and thermal power stations. Projections for impacts on hydropower and bioenergy resources are more varied. Key uncertainties and gaps remain due to the variation between climate projections, modelling limitations and the regional bias of research interests. Priorities for future research include the following: further regional impact studies for developing countries; studies examining impacts of the changing variability of renewable resources, extreme weather events and combined hazards; inclusion of multiple climate feedback mechanisms in IAMs, accounting for adaptation options and climate model uncertainty.

**Electronic supplementary material:**

The online version of this article (10.1007/s10584-018-2265-4) contains supplementary material, which is available to authorized users.

## Introduction

Major decarbonisation of the energy system has a large part to play in climate change mitigation (Bruckner et al. 2014). However, components of the energy system are also affected by climate change itself, via long-term changes in climate parameters, variability and extreme weather events (Field et al. 2014). In this fast-moving field of research, it is vital that we take stock of the literature quantifying the impacts, assess how that understanding is being used in energy system models and identify key research priorities.

Climate change impacts are expected throughout the energy system. On the demand side, the balance of heating and cooling demand patterns is changing due to rising temperatures. On the supply side, impacts include changes to the averages and variability of wind, solar and hydropower resources; the availability of crops for bioenergy feedstocks; costs and availability of fossil fuels due to melting sea ice and permafrost; the efficiency of PV panels, thermo-electric power plants and transmission lines due to rising temperatures; technology downtime due to changes in the frequency and intensity of extreme weather events (Ebinger and Vergara 2011).

These physical effects have implications for the reliability, cost and local environmental impacts of energy supply. Furthermore, some impacts may result in an increased use of fossil fuels or reinforced infrastructure, and thereby increase greenhouse gas (GHG) emissions; for example, reductions in the efficiency of power stations, reductions in renewable energy resources or increased risks of storm damage to coastal infrastructure. These would undermine efforts to decarbonise the energy sector. To ensure mitigation and adaptation options can be comprehensively examined, it is therefore imperative that climate change impacts are thoroughly accounted for in the models which are used to examine the feasibility, costs and implications of energy system decarbonisation pathways. Further research into climate impacts on the energy system and their inclusion in cross-sectoral integrated modelling is highlighted in the Fifth Assessment Report of the Intergovernmental Panel on Climate Change (IPCC AR5, Clarke et al. 2014).

A number of previous reviews examine elements of this topic (Mideksa and Kallbekken 2010; Ebinger and Vergara 2011; Schaeffer et al. 2012; Arent et al. 2014; Stanton et al. 2016; Chandramowli and Felder 2014; Ciscar and Dowling 2014). These focus largely on the climate change impacts on technologies currently in use and include relatively little discussion of the impacts on technologies which may become important in the future (e.g. bioenergy, carbon capture and storage), the levels of agreement between the results of different studies or the relative importance of impacts within regional energy systems. Comments on the strengths and weaknesses of the models used and the associated confidence that can be assigned to the results are also limited.

To facilitate the further inclusion of climate change impacts in integrated assessment models of the electricity or energy system (IAMs), we aim to address these gaps by examining the following research questions: (i) In which areas does the literature largely agree on the direction and magnitude of the impacts? (ii) Which elements are not well covered in the literature? (iii) What are the key reasons for disagreement between results? (iv) How have climate change impacts been incorporated in IAMs? (v) Which research areas should be prioritised?

This paper provides an updated, extended review of the literature on supply-side climate change impacts. Methods to incorporate impacts on heating and cooling demand in IAMs, mainly with heating and cooling degree days, have already been demonstrated by several IAMs (see OR2). So we focus here on supply-side impacts: on primary resources, generation technologies and energy transport. To examine the robustness of trends indicated in the impacts literature, we examine the levels of agreement between studies and the extent to which the system elements and regions of the world are covered (Section 3). As climate change hazards, physical and operational resilience and adaptation capability vary significantly around the world, findings from impacts literature cannot be automatically applied across regions. We discuss the principal sources of disagreement between studies (Section 4) and the ways in which climate impacts have been incorporated into IAMs (Section 5), so that we can finally prioritise the remaining research gaps (Sections 6 and 7).

## Literature review method

For this review, we define ‘impact studies’ as those which model the physical effects of changing climate on one or more elements of the energy supply system, such as a primary resource or generation technology. We define IAMs as those which represent interactions between the wider electricity or energy system and some part of the earth system such as hydrology or climate.

A semi-systematic search method was employed. Supply-side impact studies were identified via the references of previous reviews on this subject (Mideksa and Kallbekken 2010; Ebinger and Vergara 2011; Schaeffer et al. 2012; Chandramowli and Felder 2014) and a SCOPUS search using combinations of keywords relating to climate change, impacts and energy. From those search results, papers were selected providing they were published in English in a peer-reviewed journal since 2000 and focussed explicitly on physical impacts on at least one element of the energy system. For bioenergy, studies referring specifically to dedicated bioenergy crops were included. Studies employing both detailed climate change scenarios and fixed temperature/precipitation inputs were included. A database of the 106 supply-side impact studies, with details of their authors, subjects, modelling techniques and key findings, is presented in the Online Resource 1 (OR1). Studies which have incorporated one or more supply-side climate change impacts in IAMs were identified during the search described above and using additional search terms relating to electricity and integrated models. This method aimed to thoroughly identify the key literature, while remaining flexible and allowing for some judgement on the importance of various elements.

## Summary of impact studies

Table 1 summarises the impacts described in the reviewed studies. Elements of the energy system are affected by changes to average climatic conditions, the variability of conditions and the frequency and intensity of extreme weather events. Impacts studies on wind and hydropower resources dominate the literature, followed by bioenergy resources and the performance of thermo-electric power plants (TPPs). Impacts of gradual changes to climatic parameters such as temperature and precipitation have been studied more than changes to extreme weather events, as existing tools have limited ability to capture extreme events.

**Table 1:** Summary of impacts literature

System element		#Papers	Temperature		Precipitation		Windiness		Cloudiness	Heatwaves and droughts	Extreme winds	Extreme precipitation and floods
			Δ Avg	Δ Var	Δ Avg	Δ Var	Δ Avg	Δ Var	Δ Avg and Var	Δ Freq. and intensity	Δ Freq. and intensity	Δ Freq. and intensity
Hydro	Resources	33	XX	XX	XX	XX				x		XX
	Design/operations	8				XX				x		XX
Wind	Resources	34					XX	XX			XX	
	Design/operations	4					XX				XX	
Solar	Resources	5							XX	x	x	
	Design/operations	3	XX		x		x					
Wave	Resources	4					XX	XX				
	Design/operations	0										
Bioenergy	Resources	20	XX	XX	XX	XX				XX	XX	x
Fossil/nuclear fuels	Resources	0	x		x					x	x	x
TPPs	Efficiency	7	XX		XX							
	Operations	13	XX	XX	XX	XX				XX		
	Damage	1									XX	XX
Transmission and pipelines	Efficiency	2	XX				XX			x		
	Damage	2					XX				XX	x

*XX* indicates that at least one quantitative study was found examining the impact of this climatic parameter on this element of the energy system. *x* indicates that this impact is mentioned, but no quantitative studies were found. *#Papers* indicates the numbers of reviewed papers which include a quantitative study of impacts on each element of the energy system. Note, these sum to more than the total papers (106) as several examine more than one impact. *Avg* refers to changes in mean values; *Var* refers to seasonal and inter-annual variation and changes in seasonality

The reviewed studies employ a wide range of models and scenarios. See further discussion of modelling approaches, results and adaptation options in OR2. Across these, the literature indicates the following trends.

### Hydropower

Global studies differ in their projections of impacts on hydropower generation due to rising temperatures and changing precipitation patterns: Hamududu and Killingtveit (2012) and Turner et al. (2017) project that climate change will have little effect on total global resource potential, even in high emissions scenarios, whereas van Vliet et al. (2016) project a decrease of global hydropower capacity of up to 6.1% under RCP8.5 in the 2080s. Hamududu and Killingtveit (2012) also project small changes at local level (~ ± 1%), whereas van Vliet et al. (2016), Turner et al. (2017) and various regional studies (see OR1) project potentials will increase by 5–20% for most areas at high latitudes (Canada, Russia, northern Europe, northeast China) and decrease by 5–20% in regions such as southern Europe, southern USA, southeast China and southern South America.

### Wind

Reviewing wind impacts literature, Pryor and Barthelmie (2010) found average wind speeds around Europe and North America would remain within ± 15% of current values by the end of the century. This limit has since been revised up to ± 20% (Tobin et al. 2015; Davy et al. 2017) and ± 30% (Carvalho et al. 2017). Limited studies suggest there will be no significant change in wind resource over China (Chen et al. 2012) or Southern Africa (Fant et al. 2016).

### Solar

Climate change projections tend to agree that cloud cover will decrease in low- to mid-latitude regions (Patt et al. 2013); however, increases in solar resource will often be counterbalanced by decreasing efficiency due to rising temperatures. As such, regional studies tend to project changes in solar generation of less than ± 10% by the end of the century (Crook et al. 2011; Gaetani et al. 2014; Panagea et al. 2014).

### Wave

Wave resource is potentially affected by changes to wind patterns and sea level rise. However, limited studies project no change to wave generation in the Persian Gulf (Kamranzad et al. 2015) or Menorca (Sierra et al. 2017) and less than 3% in the UK (Reeve et al. 2011).

### Bioenergy

Both average yields and the areas suitable for growing bioenergy crops are affected by rising temperatures and changing precipitation patterns. Broadly, yields are expected to increase at high latitudes and decrease at low latitudes, for example by as much as − 16% and + 28% in the 2050s (Haberl et al. 2011), though studies note particularly large uncertainties regarding the importance of technological development and CO_2_ fertilisation (Haberl et al. 2011; Cosentino et al. 2012). This is expected to shift the suitable land northward for several crops (e.g. various flex food-energy crops in Europe (Tuck et al. 2006), switchgrass in the USA (Barney and DiTomaso 2010) and miscanthus globally (Hager et al. 2014)).

### Thermal power stations

Rising temperatures are expected to reduce power plant output by approximately 0.4–0.7% per degree due to reduced thermal efficiencies (Chuang and Sue 2005; Durmayaz and Sogut 2006; Linnerud et al. 2011; Ibrahim et al. 2014). Reduced water resources for cooling are expected to cause power stations to reduce load or shutdown. For example, an annual mean temperature rise of 3 °C could cause daily reductions of generation capacity of up to 36% in Germany (Koch et al. 2014). While some regions are expected to experience increased capacity under climate change (India and Russia), global annual thermal power plant capacity is likely to be reduced by 7–12% in the mid-century (van Vliet et al. 2016).

### Transmission lines

Limited studies indicate that rising temperatures will reduce the transmission capacity of overhead lines: this risk is expected to be low in the UK (Cradden and Harrison 2013), but may be significant in the USA at times of peak summer demand with reductions of up to 5.8% (Bartos et al. 2016). Increasingly, frequent storms are projected to present mostly low risks to powerlines in the UK (McColl et al. 2012), but may increase reliability indices by up to 30% in parts of Russia (Tyusov et al. 2017).

Certain impacts are less well covered in the literature. Although many papers refer to increasing damage to bioenergy crops and energy infrastructure due to storms (extreme winds, floods, landslides), very few studies were found to quantify these (one for bioenergy, three for infrastructure). No quantitative studies were found on changing maintenance requirements for solar technologies or the availability and costs of fossil fuel resource extraction as sea ice and permafrost melt. As shown in Table 2, the most studied impacts are for hydro and wind power in Europe, followed by North America. Four non-regional studies were identified examining thermodynamic behaviour of nuclear power stations, and the rating and structural failures of overhead lines. Impacts in Australasia, Asia (other than China) and Africa are under-represented. Approximately two thirds of the studies included in this review cover a sub-country, country or multi-country region, while the others are global or plant level.

**Table 2:** Summary of regional coverage

System element	Global	Europe	Asia	Africa	North America	Central and South America	Australasia	No region
Hydro	3	10	5	3	8	4		
Wind	1	17	7	3	8	2		
Solar		6	1	3	1	1	1	
Wave		3	1					
Bioenergy	2	8	3		6	1		
Fossil/nuclear fuels								
TPPs	1	12			1	1		2
Transmission and pipelines		1			1			2
Total	7	57	18	9	25	9	1	4

*#Papers* indicate the numbers of reviewed papers which cover each geographic region and energy system element

Potential for adaptation to climate change is covered inconsistently. For example, several authors comment that negative impacts on wind and solar generation and infrastructure will be insignificant because rapid development and relatively short lifetimes of these technologies allow adaptation through technological upgrades and siting (e.g. Ebinger and Vergara 2011). Careful siting of new thermal power stations may also be possible to mitigate risk of water shortages. However, most power plants are currently situated where decreasing mean annual streamflow and strongly increasing water temperatures are projected (van Vliet et al. 2016) and water competition may be exacerbated in the future by increased deployment of carbon capture and storage and biomass crops (e.g. Chandel et al. 2011; Byers et al. 2016). For siting to be considered a feasible adaptation solution at the local level for any technology, high-resolution national impact studies and communication with industry partners are required. For hydropower, design changes and dam management may also provide adaptation options (Vicuña et al. 2011). van Vliet et al. (2016) note that an increase in plant efficiency of approximately 10% would offset the mean annual negative impacts for most regions (greater improvements would be required to offset monthly impacts) but no financial cost is given for this type and level of adaptation. No quantitative studies were found to examine other adaptation options, such as changing water dispatch patterns or regulations on cooling water discharge temperatures, or strengthening generation or transmission infrastructure.

As impact studies generally examine one or two elements of the energy system in isolation and there are gaps in the regional coverage of the literature, it is unclear whether the degree to which different impacts have been studied is representative of the impacts’ perceived importance, or rather the current state of modelling ability. Relative importance can be assessed most simply by considering the percentage share of generation technologies in different regions, as done for hydropower by Turner et al. (2017). As supply technologies play different roles in the system (e.g. base or peak load generation) and may interact through water use, understanding of the relative importance of impacts and their combined effects may be improved by the use of combined modelling approaches (e.g. Rübbelke and Vögele 2013; van Vliet et al. 2016) and by the integration of several climate change impacts in models of the wider electricity and energy systems.

## Sources of disagreement

Differences between impact studies appear to be due primarily to two factors: the climate projections used as inputs to the impact models and impact model assumptions.

Many studies depend heavily on the modellers’ choice of GCM(s), especially those for which precipitation is a main driver. For example, various studies note that projections for hydropower and bioenergy resources remain highly uncertain in some regions due to disagreement between GCMs (Hamududu and Killingtveit 2012; De Laporte et al. 2014; Labriet et al. 2015a). As shown in the IPCC AR5 WGI (Stocker et al. 2013), the variation in GCM precipitation results is high over large parts of North and South America, Africa, southern Europe, southern Asia and Australia, especially under the lower emissions scenario. Modelling uncertainty is also important for wind and solar studies. Examining an ensemble of 10 GCMs, Karnauskas et al. (2017) conclude that results for some regions (tropics and southern sub-tropics) are only robust for the later part of the century under the higher emissions scenario. Fant et al. (2016) note the high uncertainty in cloud cover projections may account for the low number of solar impact studies. As well as preventing robust impact predictions, climate modelling uncertainty can preclude meaningful evaluation of adaptation options (Donatelli et al. 2015). Where there is a high variation between GCMs, impact models can be driven with projections representing the full range of GCM results to represent the uncertainty, as was done for hydropower in Ecuador by Carvajal et al. (2017).

Uncertainty due to modelling assumptions is especially clear for bioenergy impact studies. These results vary due to their treatment of factors such as technological development, levels of agricultural input such as pesticides and fertilisers, irrigation, climate change impacts on water resources and CO_2_ fertilisation (e.g. Brown et al. 2000; Haberl 2011). Asseng (2013) and Rosenzweig et al. (2014) each performed standardised model comparisons to explore these differences. Asseng (2013) concluded that a greater proportion of the uncertainty in impact projections was due to differences between crop models than between the climate models. Rosenzweig et al. (2014) found crop model variation is highest in the middle latitudes and agreed that uncertainties related to the representation of carbon dioxide, nitrogen and high temperature effects are key. Both note that reducing these uncertainties is vital in order to facilitate adaptation responses.

## Representation in IAMs

Climate change impacts have been represented in IAMs most commonly as economic damage functions, for use in mitigation-adaptation cost benefit analysis. Comparing climate damage functions in several top-down IAMs, Warren et al. (2006) and Ortiz and Markandya (2009) both note their uncertainties are high. This is because they tend to be based on a small number of empirical studies and because empirical studies are often regional or sector specific whereas IAMs are often global, so relationships must be extrapolated across regions with varied physical and economic structures and constraints.

Including physical technology-level impacts has been identified as a research gap by the IPCC Working Group III (Clarke et al. 2014). This is required for cost-effectiveness analysis of climate stabilisation paths because, as described above, climate change can affect the availability of low-carbon supply options, necessitate reinforcement of the supply system and impact GHG emissions (Fisher-Vanden et al. 2013). Modelling physical impacts can also allow examination of their relative importance compared to socio-economic drivers (e.g. Isaac and van Vuuren 2009) and exploration of adaptation options to reduce impacts on the whole system rather than an individual element. For example, by allowing and restricting various combinations of other generation technologies (e.g. natural gas), Parkinson and Djilali (2015) demonstrate that wind energy with storage and/or interregional transmission could be combined in a strategy of co-beneficial mitigation and adaptation.

Table 3 summarises IAMs which have incorporated supply-side climate impacts. Changes to hydropower resource have been incorporated most; the high interest in this technology is likely due to the dominance of hydropower in some countries as a baseload electricity generator, its long project lifetime and the clear weather-dependence of the resource. Note, other studies have incorporated demand impacts alone, mostly using heating and cooling degree days (Isaac and van Vuuren 2009; Labriet et al. 2015b; McFarland et al. 2015). A few have included multiple demand and supply-side impacts together, allowing examination of their relative importance and how their combined effect might increase or mitigate pressure on the system.

**Table 3:** Models which include physical supply-side climate impacts

Model		Key papers	Impacts modelled
*TIMES* - Energy system optimisation	Portugal	Cleto et al. (2008)	Hydro resource
		Teotonio and Rodríguez (2017)	
*SUSHI-O* -Generation system operation simulation	Brazil	de Lucena et al. (2010)	Hydro + biofuel resource
*MARKAL* - Energy system optimisation	Norway	Seljom et al. (2011)	Heating/cooling demand Wind + hydro resource
*ICEM* - Community-scale energy system optimisation	No region	Cai et al. (2012)	Hydro + wind + solar resource
*POLES* - Energy system simulation	Europe	Dowling (2013)	Heating/cooling demand
			TPP + solar PV efficiency Wind + hydro resource
			Hydro installed capacity
		Mima and Criqui (2015)	Heating/cooling demand
			Nuclear + TPP cooling Hydro resource
*LPJmL + GCAM* - Crop growth model and dynamic recursive model	Global	Kyle et al. (2014)	Bioenergy resource
Coupled hydrological and power system model	Iberian peninsula	Pereira-Cardenal et al. (2014)	Heating/cooling demand
			Hydro resource
*TIAM-WORLD + PLASIM-ENTS* - Coupled energy system optimisation model and climate emulator	Global	Labriet et al. (2015a)	Heating/cooling demand
			Hydro resource
Electricity system planning framework	British Colombia	Parkinson and Djilali (2015)	Heating/cooling demand
			Hydro resource
Hydro-thermal power scheduling optimisation	Brazil	de Queiroz et al. (2016)	Hydro resource
Coupled water availability and electricity production cost simulation model	Western USA	Voisin et al. (2016)	Hydro resource
			TPP generation
*LEAP + WEAP* - Coupled energy system and water resource simulation models	Zambezi basin	Spalding-Fecher et al. (2017)	Hydro resource
*HiREPS* - Dynamic simulation and optimisation	Austria and Germany	Totschnig et al. (2017)	Heating/cooling demand
			Wind + PV + hydro resource
			TPP cooling
Coupled dynamic hydropower scheduling model and Real Time Pricing model	Norway, Sweden, Finland	Hilden et al. (2017)	Hydro resource
Decision scaling framework with ICHYMOD hydrological model	Northern Italy	François et al. (2018)	Hydro resource

In most of these studies, impacts were simulated exogenously from the energy system model. More thorough representation has previously been limited by challenges in finding suitable climate data, translating climate data into a form that is relevant for the energy system, and lack of detail in energy system models (Dowling 2013). Incorporating physical impacts endogenously could enable the researchers to more easily examine the full feedback mechanisms. For example, by iteratively exchanging data between an energy system model and climate emulator, Labriet et al. (2015a) model how changes to demand patterns and hydropower potential induce changes to the GHGs emitted by the energy system, which in turn modifies the climate change pathway.

Representing climate impacts in wider energy system models requires careful consideration of temporal and spatial resolution and extent. For example, for energy system optimisation modelling, a time horizon of a few decades may be chosen to limit the impact of growing technology and cost uncertainties. However, a longer time horizon may be desirable to examine the larger impacts of climate change or the importance of technology lock-in (Parkinson and Djilali 2015). Some impact models require high-resolution input data, such as local topographic features in process-based hydrological models. Due to computational expense, these are often run at regional scales, making it more difficult to combine them with global-scale energy system models (Parkinson and Djilali 2015). Global models may use simpler simulations and cover a much larger extent but include less detail on land characteristics and processes (e.g. Labriet et al. 2015a). Finally, global IAMs generally represent the world as approximately 10–30 regions only, so climate impact data must be aggregated to this scale. The variations in impacts within these regions can present problems for this aggregation.

As for impact studies, several IAM authors (e.g. de Lucena et al. 2010) note their results are highly dependent on the climate model used as the driver. To reduce the impact of the selection of a single climate projection, some studies use an ensemble mean (e.g. Seljom et al. 2011; Parkinson and Djilali 2015). However, little discussion is included evaluating the strengths or weaknesses of these approaches. Mima and Criqui (2015) examined this uncertainty by running the POLES model with 10 climate model projections for a single emissions scenario, noting the high variation prohibits decision-making in some cases.

Using full climate models in conjunction with energy system models is computationally expensive (Labriet et al. 2015b), so some use a climate simulator model. Endogenous representation of climate change impacts in IAMs could be limited by this representation of climate change. In a comparison of how well seven well-developed IAMs simulate climate change, van Vuuren et al. (2011) note large variations in the steps by which IAMs link GHG emissions to temperature change: the equilibrium and transient climate change calculations, radiative forcing response to changes in CO_2_ concentration and the behaviour of the carbon cycle. However, the comparison found that the climate change results of most IAMs are within the range of the outcomes of the more complex models, indicating that climate change simulation should not prevent further work on incorporating impacts into IAMs.

To address the extensive uncertainties involved in climate change impact modelling, and the difficulties involved in quantifying them, multi-model comparisons can be used. For example, McFarland et al. (2015) incorporated demand-side climate change impacts in three US power sector models aiming to increase the robustness of the results. This study identified that the main source of difference between the models was the underlying data used for calibration, though the authors did not narrow the range of results based on this conclusion. Going forward, multi-model comparisons would be useful to explore the differences between parametric and model structure uncertainty.

## Research gaps

A number of key gaps remain in the literature on supply-side impacts.

### Energy system elements

There are relatively few studies on wave power and none on tidal power, likely due to their low market share, and solar power, likely due to the rapid growth of the industry and assumed flexibility of siting options. Quantitative modelling of impacts on transmission and coastal infrastructure is also limited and a large-scale analysis of impacts on second-generation bioenergy resources is lacking.

### Climate elements

Impacts of extreme weather events under climate change scenarios have been less extensively studied than gradual changes. This is probably due to the larger uncertainty in this area of climate science, where the IPCC AR5 gave a low confidence assessment on the future trends of droughts and floods (Stocker et al. 2013). Furthermore, though studies can examine the impact of past events using historic data, the application of these findings to projections of future extreme events may be difficult due to the high number of variables involved. As demonstrated by US and European outage events, power station shutdowns caused by droughts and heatwaves can have dire consequences (Lagadec 2004). Significant work should be undertaken to understand their frequency, so that contingency plans and warning systems may be implemented. Impacts of combined or consecutive events and the cascading effect of extreme weather events through the transport, telecoms and water sectors are important but still understudied (Ciscar and Dowling 2014). Studies examining the impact of changes to the variability of climatic conditions are also limited, though this may be very important for renewable integration (Hueging et al. 2013; Santos et al. 2015).

Energy-water interactions are important for many parts of the energy system: hydro, bioenergy and fossil fuel resources, thermal power plant efficiency and availability, CCS. Energy-water nexus modelling for these elements on a large geographic scale has been limited as it presents particular challenges related to the importance of local hydrological systems and due to the high variation in the precipitation results from different GCMs (Stocker et al. 2013).

### Regions

As described in Section 3 and consistent with the summary in IPCC WGII (Field et al. 2014), regional studies are still scarce for developing countries, indicating that data availability and research funding are likely significant factors in the choice of study area. In general, comments on the extent to which studies are applicable to the wider industry and geography are limited but would be useful to aid the prioritisation of further research. Furthermore, few climate change impact studies are performed at the global scale, as regional studies have the advantage of being able to use higher resolution data. Further global studies and regional studies for developing countries are needed to facilitate the inclusion of the impacts in global IAMs.

### Uncertainties

More detailed examination of the effects of climate, policy and impact uncertainties on modelling outcomes is needed (Chandramowli and Felder 2014). The use of a climate model ensemble mean can reduce the uncertainty due to differences between climate models; however, it does not address the uncertainty analytically or allow the modeller to quantify it. It may also hide the effect of extreme but uncommon events. Further sensitivity assessments using the full range of GCMs (such as Carvajal et al. 2017) and intermodel comparisons, in the style of the ISI-MIP project (Warszawski et al. 2014), are needed in order to address these uncertainties more explicitly.

### Relative importance

Few systematic comparisons of the relative importance of the impacts were found; regional assessments of this kind are important in order for energy system planners or operators to prioritise investment, for example in strengthening the infrastructure or reducing energy service demands. It could be that these assessments are carried out in government or industry reports rather than peer-reviewed literature. Inclusion of climate change impacts in IAMs can be a way of identifying their relative importance in the system context.

### Inclusion in IAMs

Only a few studies have integrated technical supply-side climate change impacts in a bottom-up IAM, likely due to the technical and data challenges described in Section 5. Those which did indicated that the impacts are significant when considered in combination (e.g. Dowling 2013; Pereira-Cardenal et al. 2014). Therefore, it seems clear that models examining long-term energy system pathways should incorporate climate change impacts on as many elements as are relevant for the regions covered, in order to capture any ways in which they balance or combine with each other.

## Recommendations

To facilitate further integration of climate change feedback in sectoral models and IAMs, the following key areas should be prioritised in future research.

First, further impact studies should focus on the energy system elements in developing countries. As vulnerability of infrastructure and operational systems vary significantly between regions, these are needed for both effective system planning locally and effective representation in large-scale IAMs.

Second, further studies are needed on the technical and cost impacts of altered variability of renewable resources, and the impacts of extreme weather events on all elements of the energy system. As the impacts on individual system elements become better understood, research should turn to the combined effects of simultaneous or successive impacts, such as water shortages at times of high cooling demand, or storms hitting already weakened infrastructure. These could entail probabilistic modelling approaches.

Third, IAMs should be developed to incorporate multiple climate change impacts on the demand and supply side at the same time. Technological and behavioural adaptation options and water competition should be represented to improve the comprehensive examination of the costs, feasibility and optimal timing of energy system decarbonisation pathways. As the variation between GCMs is a key uncertainty, methods should be developed to better reflect the range of climate projections, beyond the use of a climate model ensemble mean.

## Electronic supplementary material

ESM 1 (XLSX 1779 kb)

ESM 2 (DOCX 95 kb)
