# Supplementary material for: Climate change impacts on the energy system: a review of trends and gaps
Source: Clim Change. 2018 Aug 6;151(2):79–93. doi: 10.1007/s10584-018-2265-4 (PMC6404738; doi:10.1007/s10584-018-2265-4)
Supplement: Supplementary file 2 — (DOCX 95 kb) [file 10584_2018_2265_MOESM2_ESM.docx]

# Online Resource 2: Impact results and models

# Hydro power

Climate change affects hydropower resources via annual river-flow, flow seasonality and flow variability. These are driven by changes to average and extreme precipitation patterns and temperature, especially in river catchments which rely on snow- or glacial- melt. Increased runoff will not always increase energy generation as some plants will operate sub-optimally under altered in-flow conditions (Mukheibir 2013).

Global, regional and plant-level models are used in hydropower studies. Global studies are generally not able to account for plant-specific factors such as turbine type, efficiency or operational adaptation and generally use low resolution climate data due to computational limits (Hamududu & Killingtveit 2012). Basin-level studies clearly demonstrate the importance of modelling local hydrological effects; for example, Vicuña et al. (2011) project that the lower elevation power plants in California will experience a greater change in runoff seasonality, whereas in the Swiss Alps Majone et al. (2016) project the greater change for the power plants at higher elevation.

Three key global studies assess these impacts: Hamududu & Killingtveit (2012) use an ensemble of simulations of regional hydrological runoff models; van Vliet et al. (2016) use a physical hydrological and water temperature model coupled with hydropower and thermoelectric power models; (Turner et al. 2017) use a coupled hydrological and dam model. Hamududu & Killingtveit (2012) and (Turner et al. (2017) predict that climate change will have little effect on total global resource potential but significant effects at regional and country level for the A1B and RCP8.5 scenarios. However, van Vliet et al. (2016) project a decrease of global hydropower capacity of up to 6.1% under RCP8.5 in the 2080s.

Regional studies examining impacts on hydropower resources (several summarised by Mukheibir 2013) tend to agree with the results of the global studies, predicting increases in hydropower potential for most areas at high latitudes (Canada, Russia, northern Europe e.g. (van Vliet et al. 2013, Tyusov et al. 2017) and tropical regions (central Africa, southern Asia) and decreases in potential for other regions (e.g. Harrison & Whittington 2002, de Lucena et al. 2009, Pašičko et al. 2012, Spalding-Fecher et al. 2016).

A key uncertainty in hydropower resource modelling is the projection of temperature and precipitation, which drive in-flow. To reduce this model uncertainty, several studies employ an ensemble mean of climate models, however the robustness of this method is often not discussed. This issue of variation between climate models is discussed in the article Section 4.

Adaptation options for hydropower plants include both design changes and operational changes with the use of dams (Vicuña et al. 2011). Van Vliet et al. (2016) note that although most hydropower plants are situated in regions where decreasing streamflow is projected, an increase in plant efficiency of approximately 10% would offset the mean annual negative impacts for most regions; greater improvements would be required to offset the monthly impacts. However, no financial cost is given for this type and level of adaptation. Some studies (e.g. Gaudard et al. 2014) consider the impacts of climate change on both hydropower resource and electricity prices, pointing out that these will combine in determining the optimal hydropower management strategies.

# Wind power

Climate change may affect the geographic distribution of wind resource through changes to the annual and seasonal mean wind speeds, and air density. Extreme winds, icing of turbine blades and sea ice/permafrost can impact the required design, operations and maintenance of wind farms (Pryor & Barthelmie 2013). In some regions, climate change will also cause changes to the inter- and intra-annual variation of wind speeds which will significantly impact the availability and management of wind generation (Hueging et al. 2013, Santos et al. 2015).

As wind speed distributions are highly dominated by local climate effects (e.g. coastal or inland thermal drivers) and land features, wind resource studies tend to employ downscaled climate model projections and are mostly conducted at regional levels (Pryor & Barthelmie 2010). Relatively few studies consider impacts on offshore wind regimes.

The Pryor & Barthelmie (2010) review concludes that literature indicates it is unlikely that mean wind speeds and energy density in Europe and North America will change by more than ±15% during this century, which is within the range of current inter-annual variation. This limit was revised up slightly by Tobin et al. (2015), which found that for most of Europe, with the A1B emissions scenario, wind power resource will remain within ±20% of current values by the end of the century, with a tendency towards increases over northern Europe and decreases over southern Europe, and again by Carvalho et al. (2017) who project changes of +30% and -30% in North-Central Europe and Eastern Europe respectively. Limited regional studies suggest there will be no significant change in wind resource over China (Chen et al. 2012) or Southern Africa (Fant et al. 2016). No local wind generation impact studies were identified for other Asian, South American, Australasian and African regions. Most recently, Karnauskas et al. (2017) projected wind production with an ensemble of 10 GCMs and found robust decreases in wind power across the mid-latitudes of the Northern Hemisphere, and increases across the tropics and Southern Hemisphere but with significant spread between GCMs and emissions scenarios.

Due to the non-linearity of the wind energy density equation and the turbine power curve, the electricity generation from a turbine is dominated by the upper percentiles of the wind speed distribution and therefore changes in mean wind speed alone are insufficient to represent changes in production (Pryor & Barthelmie 2013). Extreme winds can also impact the required design of wind turbines and their operations and maintenance. For example, for Indian offshore wind sites, Deepthi & Deo (2010) project that the 100-year return wind speed could increase by up to 74% towards the end of the century in the A2 scenario and Kulkarni et al. (2014) project that the turbine design wind speed could increase by up to 17% in RCP4.5 by 2035.

As for hydropower, the variation between climate models is a key source of uncertainty in wind resource projections. Several authors note the high uncertainty in their results: De Lucena et al. (2010) remark that the findings should be considered a possibility rather than a projection due to the use of a single GCM and national wind database; Tobin et al. (2015) comment that their predictions may be useful in optimising the energy mix long-term but should not be considered for individual projects in the coming decades.

Adaptation options for wind power include wind farm siting (prioritising locations where long-term average wind speeds are projected to remain the same or increase), and changes to design and operations such as the use of heated turbine blades and dynamic control systems (Pryor & Barthelmie 2013). The relatively short lifetime of wind power projects and the fast improvements in technologies of wind technologies suggest that negative impacts are likely to be designed out (Ebinger & Vergara 2011). However, for system planners or project developers to account for climate change impacts in their wind farm siting or project design decisions, consensus between studies and quantitative uncertainty estimates would be necessary.

# Solar power

Climate change impacts solar generation by altering cloud patterns and ambient temperature. The efficiency of photovoltaic cells (PV) decrease almost linearly with increasing temperature while the output of concentrated solar power (CSP) increases. CSP output increases almost proportionally with increasing direct irradiance and PV output increases almost proportionally with total irradiance except under low light levels (Crook et al. 2011). Changes to strong winds, hail and dustiness may also impact required design or maintenance of solar technologies.

Just a handful of regional studies examining climate change impacts on solar generation have been performed, covering mainly Europe and Africa, making it difficult to judge significant trends. No global studies were identified in this review. Studies employ the temperature, cloud cover and irradiance or insolation results of climate models. Climate change projections tend to agree that cloud cover will decrease in low to mid-latitude regions (Patt et al. 2013), however, local impacts on cloud cover remain highly uncertain. Along with the rapid roll-out of solar technologies, this uncertainty may account for the small number of solar impact studies (Fant et al. 2016).

Generation impact studies tend to account for changes to both the solar resource and the technology efficiency, which often change in opposite directions. For example for Greece, Panagea et al. (2014) project a significant increase of mean annual temperature, which would decrease the efficiency of PV systems, but would be outweighed by the significant increase in total irradiation and so predicted an increase in energy output by the end of the century of 4%. For the B2 scenario, Gaetani et al (2014) project that PV output will decrease 7% in Eastern Europe and Northern Africa but increase by 10% in Western Europe and the Eastern Mediterranean by the end of the century. This is slightly higher than the results of Crook et al. (2011) who projected increases of 3-4% in Spain, Germany and China and decreases of 2-6% for the US and Saudi Arabia, for the A1B scenario, depending on the GCM used. Most recently, Fant et al. (2016) project close to zero change in the long-term mean global horizontal irradiance and low probability of significant changes to the extremes for southern Africa.

Several papers remark that strong winds can damage solar energy structures, that dust and sand deposits can significantly increase the need for cleaning, and that the frequency and intensity of these events may increase with climate change (Patt et al. 2013, Arent et al. 2014), however no studies were found which quantify these impacts on generation under climate change scenarios.

It is generally agreed that the relatively small predicted impacts, the short life-time of solar installations and the fast rate of technological development and cost reductions mean negative impacts of climate change on solar generation will be designed out and are no cause for concern (Arent et al. 2014). Even so, it may be beneficial to make PV panels more heat resistant and improve CSP cooling systems (Patt et al. 2013).

# Wave and tidal power

Wave power resource is affected by changes to wind patterns and sea level rise. Just a few studies considering these impacts were identified. No quantitative studies of the effect of climate change on tidal power were identified. The limited focus on wave and tidal power in this field of literature appears to reflect the small market share of these technologies.

Using RCM wind data and the WW3 wave model for a wave energy converter off the coast of Cornwall, UK, Reeve et al. (2011) predicted that wave power would increase by 2-3% in the A1 scenario but decrease by 1-3% in the B1 scenario, suggesting that better climate change mitigation would decrease the wave resource. Using downscaled wind data from a single GCM to drive the SWAN wave model for the Persian Gulf, Kamranzad et al. (2015) projected no significant change in the 30-year average wave energy in any of the scenarios tested (A2, B2, A1B). However, the study also found that climate change may impact the maximum annual wave energy and other characteristics of the wave resource (significant wave height and peak period), which may be significant in the selection of wave energy converters.

# Bioenergy

Bioenergy crop resources are affected by changes to seasonal patterns of temperature and precipitation as well as extreme events such as heatwaves, droughts and hurricanes. (Chum et al. 2011). The impact of increased atmospheric CO_2_ concentration itself is uncertain as it increases photosynthesis and the water efficiency of plants but may also favour certain pests and diseases (Gomall et al. 2010, FAO 2016).

Studies estimating biomass crop resources and bioenergy potential and the impacts of climate change are mainly conducted at national or regional level. The studies’ temporal and spatial scales are often set by the climate models (Long et al. 2013). Insights can also be gained from literature focussing on food and fodder crops (e.g. Rosenzweig et al. 2014, Muller & Robertson 2014). Literature indicates the net impact on global resources is expected to be small but regional impacts may be significant (Chum et al. 2011).

Several studies (e.g. Barney & DiTomaso 2010, Khan & Khan 2014) use land suitability classification models, which test each land parcel against soil, terrain and climatic criteria. Tuck et al. (2006) used such a model to assess the changes in the distributions of a series of oily, starchy and solid biomass crops, projecting that in the 2080s the distribution of most crops increases in northern Europe due to increasing average temperatures, but decreases in southern Europe due to increasing droughts. This agrees with Zabel et al. (2014), who performed a similar study on the global scale for 16 food crops and found for the A1B scenario climate change will expand suitable crop land in the high northern latitudes, but decrease it in tropical regions, notably in the global south. Using a Maximum Entropy Algorithm Species distribution model, W. Wang et al. (2014) examined the relative importance of the different input parameters and found for bioenergy crops in China the variables ‘precipitations of the warmest quarter’ and ‘annual mean temperature’ are the most important climate factors.

As summarised by the IPCC AR5, results of land suitability and gridded yield models tend to indicate a trend of decreasing yields, particularly in low latitudes, with some predictions of increasing yields in higher latitudes, with southern Africa and South Asia likely to suffer the worst negative impacts. Literature indicates that ‘negative impacts [on crop yields] of more than 5% become *more likely than not*’ and from the 2080s ‘negative impacts in the tropics are *very likely*’*.* These statistics represent a summary of crop yield projections for all emissions scenarios, regions, crops and levels of adaptation (Porter et al. 2014). Note that some recent research suggests that process-driven crop yield models may underestimate the detrimental effect of very high temperature days (Schauberger et al. 2017).

Studies on the impacts of extreme weather events in climate change scenarios on bioenergy resources are limited, but the studies there are indicate that the impacts may be substantial. Stromberg et al. (2011) used historical typhoon and crop damage data and a Monte Carlo model to simulate the intensity of future typhoons and thus examine the losses of bioenergy resources in the Philippines under two climate change scenarios. From these results, total crop losses are expected to increase by 34% by 2050. Lesk et al. (2016) examined the impacts of extreme weather events by estimating the national losses of cereal crops around the world from reported historic extreme weather events. The results indicated that between 1964 and 2007 droughts and extreme heat reduced national cereal production by 9-10%, most significantly in developing countries.

Related issues around competition for land between energy crops and with food crops, forestry and conservation land as well as water competition are examined in papers such as Fuss et al. (2014). Some of these factors could exacerbate negative impacts of climate change on bioenergy resources, as it may be assumed that demand for food crops will be met before energy. Combined qualitative and quantitative methods are also used in this field. For example, Olesen et al. (2011) surveyed agriculture experts across Europe to gain understanding of their perception of climate change impacts and adaptation options. Langholtz et al. (2014) used the IPCC hazard-exposure-vulnerability risk assessment framework of to examine risks to the US lignocellulosic feedstock supply chain and found that although the increasing hazards are likely to disproportionately affect the regions of energy crop growth, several adaptation options are available.

Hertel & Lobell (2014) explain that adaptation can be driven by the market or planned at a regional or national level. For example, options using existing technologies include increasing the use of labour, fertilizers, pesticides or irrigation; research and development may be focussed on bringing more suitable cultivars to market; policy and market environments can provide services such as crop insurance which could incentivise riskier behaviour. The survey by Olesen et al. (2011) found that European farmers are already adapting to climate change impacts by changing the timing of cultivation and selecting different crop species. These authors note the importance of public/industry perception of climate change impacts, for example the survey results indicate a more negative expectation over Northern Europe than is suggested by the literature; this kind of inconsistency could well have an impact on adaptation roll-out.

# Thermal power plants

Increased water or air temperatures can reduce the efficiency of thermal power plants (TPPs). Insufficient water availability or increased water temperatures can mean power stations have to reduce their load or shut down due to physical laws or to prevent environmental regulations being breached (Schaeffer et al. 2012). Increased hail, precipitation, winds, floods and lightning cause increased physical damage to TPP structures (Sieber 2013).

Power stations’ water demand depend heavily on their cooling system. For once-through systems, it depends on the incoming water temperature, the amount of waste heat and the permissible exit water temp. For systems with cooling towers, it depends on the losses to the air, which are driven by the air temperature and humidity. A lower permissible rise in exit water temperature or greater losses from cooling towers can be compensated by a higher volume of cooling water uptake. Therefore, projections of future water demand may involve projections of electricity demand, power station stocks and air and water temperatures (Koch & Vögele 2009, Rubbelke & Voegele 2011). Due to their large water requirement, power plants with once-through cooling systems are most affected by climate change. Nuclear power plants are more vulnerable than other fuel plants as their efficiency is lower, they require more water per unit of generation and multiple stations often share cooling water supplies (Linnerud et al. 2011). Byers et al. (2014) demonstrate that the addition of CCS to thermal power stations approximately doubles their water withdrawal and consumption and note that new power stations may be encouraged to cluster in order to benefit from shared infrastructure. These two factors may worsen TPPs’ vulnerability to water shortages (Byers et al. 2016).

Impacts on TPPs have been analysed mostly at a plant-level and regional scale using a variety of tools including thermodynamic and hydrologic models (e.g. Koch & Vögele 2013), system dynamics modelling (Hoffmann et al. 2013) and econometric approaches (Linnerud et al. 2011). Theoretical models are advantageous as they can isolate the impact of climate/weather variables. Analysis using plant-level measured operational panel data (e.g. Linnerud et al. 2011) may have the advantage of the data encompassing operational adaptation measures, which may overcome the physical impacts. Wider water-energy nexus models have been used to consider the water competition between several energy technologies and other sectors on a regional basis (e.g. Flörke et al. 2011, Spalding-Fecher et al. 2016).

Plant-level models tend to agree that power stations’ power output decrease by approximately 0.4 – 0.7% for each 1°C rise in water temperature due to reduced thermodynamic efficiency (Durmayaz & Sogut 2006, Linnerud et al. 2011, Ibrahim et al. 2014). At high temperatures, this may rise to 2.3% loss per 1°C rise (Linnerud et al. 2011). Although these efficiency impacts appear small, the cumulative impact over many stations could be large, especially when combined with periods of reduced load during heat waves (Chandramowli & Felder 2014). Combining the two impacts of reduced thermal efficiency and reduced load due to water availability, van Vliet et al. (2013) project that thermal power generation will be decreased by approximately 10-14% in Spain and Bulgaria and by 2-5% in central Europe in 2031-2060 in scenarios A2 and B1, with greater reductions in summer. In a global study, Van Vliet et al. (2016) found that most thermoelectric plants are situated in areas with expected decreases in mean annual streamflow and strong water temperature increases and thus the global annual usable capacity is expected to be reduced by 7-12% in the 2050s under climate change scenarios RCP2.6-8.5.

Extreme winds and lightning storms can cause damage to cooling tower structures, tower insulation, storage tanks and, utility poles, and control equipment, while flooding can uproot tanks and cables and cause electrical faults (Sieber 2013). Very little literature projecting these impacts under climate change scenarios or indicating their relative importance were identified in this review. One example is the study by Kopytko & Perkins (2011), who assessed risk criteria for inland and coastal nuclear plants across the USA, and found that safety is the greatest concern for coastal plants and interrupted operation is more significant for inland sites.

Adaptation options for TPPs include: alterations to their cooling systems, such as installing dry cooling tower, regenerative cooling or heat pipe exchangers, recycling water or using non-traditional water sources; reinforcing structures and strengthening structural regulations; installing flood control measures such as dams and channels; and careful siting of new plants (Sieber 2013). Some power plants will be able to avoid periods of reduced load by scheduling their planned maintenance during the hottest summer months (Rubbelke 2011). As for all technologies, TPP impacts and adaptation options must be considered in combination. For example, building a new power station on the coast may reduce its vulnerability to cooling water shortages but may increase costs for flood defences (Byers et al. 2014). Testing the efficacy of various adaptation options, Van Vliet et al. (2016) found that even large improvements to TPP efficiency (e.g. 20% increase) would not be enough to offset the negative impacts due to water availability. However, large-scale switching from once-through cooling systems to recirculation systems could be sufficient and, in Africa and Australia, switching from coal to more efficient gas-fired TPPs would be very advantageous.

# Access, storage and transport of energy commodities

Climate change may have both positive and negative impacts for fossil fuel extraction: reduced sea ice may ease shipping access in the Arctic for drilling materials and liquefied natural gas but thawing permafrost is expected to threaten the integrity of oil and gas drilling infrastructure (Wilbanks et al. 2014). Climate change presents several increased risks for the industry through the occurrence of adverse weather events. Increased temperatures reduce the efficiency of steam turbines and encourage biological growth in equipment used in oil refining; a higher frequency of very hot days, heavy precipitation and landslips increases maintenance needs for roads and rail tracks used for transporting commodities; increased storms, lightning and floods increase damage to infrastructure including inland oil fields and pipelines, transport of oil and gas by shipping would be upset by increased flooding or storm surges (Cruz & Krausmann 2013). Finally, pressure in gas and oil pipelines is sensitive to temperature changes and coal stockpiles are vulnerable to increased humidity, flooding and self-ignition in heatwaves (Sieber 2013). No studies examining the change in these impacts under climate change scenarios were identified in this review. Reinforcement of infrastructure or use of heat resistant materials may reduce some of these impacts; changing the shape, coating and wind breaks around coal stockpiles can reduces losses to wind and protect (Sieber 2013).

Climate change is expected to impact both the efficiency and structural integrity of electricity lines. The current carrying capacity of electricity lines (‘rating’) decreases with increasing line temperature, which is affected by air temperature, wind speeds and solar irradiation. Increased storms are expected to cause structural damage and may require increased maintenance.

Literature on the effect of climate change on the efficiency of overhead lines (OHLs) has been mainly qualitative or has used only mean temperature projections or worst-case climate projections. Cradden & Harrison (2013) summarise the worst case projections, indicating that for each 1°C increase in ambient temperature, OHL ratings decrease by 8-14% (distribution), 2-4% (transmission) by 2099. Cradden & Harrison (2013) improved on previous studies using hourly time series data from the UK Climate Projections probabilistic climate change modelling framework to assess the effect of temperature and also solar irradiance wind conditions. The authors conclude that in a medium climate change scenario, there is a small increase in the likelihood of UK OHLs exceeding nominal ratings but the total risk is still small. Bartos et al. (2016) found significantly higher results for the USA using a combination of thermal models of representative conductors and regression analysis of historical load data, predicting that the average summertime transmission capacity reduces by 2-6% and the peak per-capita summertime load rises by 4-15% (ranges for RCP2.6-8.5).

Several studies (e.g. Musilek et al. 2009, Matko et al. 2016) consider impacts of weather events (rather than changes in typical ambient conditions) on electrical infrastructure: for example, Reed (2008) evaluated the performance of an urban power grid system during winter storms with a combined statistical-GIS method, and Winkler et al. (2010) considered the performance of topologically diverse power systems subjected to hurricanes. From this type of study, trends under climate change scenarios could be inferred, however very few papers examining these impacts under climate change scenarios were identified. One example is a UK study by McColl et al. (2012) who project the occurrence of gale, heat, lightning and flooding faults may increase but snow and sleet faults may decrease (A1B scenario). Underground cables are less vulnerable to storm and ice damage than overhead lines, however they are 5-20 times more expensive (Sieber 2013).

# Energy demand

The majority of demand-side impact studies focus on changes to demand for heating and cooling services in buildings due to rising atmospheric temperatures. Demand studies have used a wide variety of approaches including cross-sectional data, univariate time-series data and panel data (Auffhammer & Mansur 2014). Human behaviour is highly important in demand studies: responses to climate change will depend on habits and cultural expectations. Auffhammer & Mansur (2014) distinguish between the intensive margin (how consumers respond to short-term unusual weather events) and the extensive margin (how they respond to long-term changes) and conclude that studies on the intensive margin have largely used aggregated panel data and that further use of household-level data may be useful in examining behaviour.

Recent summaries of literature on the impacts of climate change on energy demands were presented by Auffhammer & Mansur (2014), Chandramowli & Felder (2014) and Brown et al. (2016). Demand-side studies largely focus on the residential sector at city or country-level, while large-scale studies applicable to large regions or globally are still lacking. Demand-side impact studies mainly focus on the residential sector and similar studies are needed for the industrial and commercial sectors (Auffhammer & Mansur 2014). These authors also identify the need for more research which includes adaptation to changes in mean conditions through technology adoption (e.g. air conditioners), more research that uses household and firm-level panel data of energy consumption during extreme weather events, and more research which considers different temperature comfort levels in different regions.

Decreases in final energy demand for cooling services and increases for heating services are expected to largely compensate each other on the global scale but may be significant at regional scale. Which impact dominates will depend on the local climate but also regional levels of economic development, fuel choices, household incomes and levels of access to heating and cooling technologies (Arent et al. 2014). De Cian et al. (2013), whose derived elasticity values are used in several other models, note that as well as the net change in energy service demand, the change in the fuel mix is also key. In many regions, heating is largely provided by primary fuels. If those fuels are replaced with electricity for cooling, then annual and peak electricity demand may well increase.

Demand-side climate change impacts have been represented in several integrated assessment models, largely using heating and cooling degree days. Isaac & van Vuuren (2009) used the H/CDDs directly as a driver in their demand calculations, while Dowling (2013), Seljom et al. (2011) and Labriet, Joshi et al. (2015) scaled their base case space heating and cooling demands by the ratio of HDDs or CDDs in the climate change and base cases. Parkinson & Djilali (2015) instead used a regression analysis of temperature and demand data. How best to derive appropriate set point temperatures for the calculation of H/CDDs from measured electricity demand data is debated, as they may be different for heating and cooling, vary between sectors and building types, and depend on cultural preferences (Brown et al. 2016). Still, using H/CDDs is a preferred method for representing this climate change impact in wider energy models as it incorporates the magnitude and time duration of the required temperature change.

# References

Arent, D.J. et al., 2014. Chapter 10 - Key economic sectors and services. *Climate Change 2014: Impacts, Adaptation, and Vulnerability. Part A: Global and Sectoral Aspects. Contribution of Working Group II to the Fifth Assessment Report of the Intergovernmental Panel on Climate Change*.

Auffhammer, M. & Mansur, E.T., 2014. Measuring climatic impacts on energy consumption: A review of the empirical literature. *Energy Economics*, 46, pp.522–530.

Barney, J.N. & DiTomaso, J.M., 2010. Bioclimatic predictions of habitat suitability for the biofuel switchgrass in North America under current and future climate scenarios. *Biomass and Bioenergy*, 34(1), pp.124–133.

Bartos, M. et al., 2016. Impacts of rising air temperatures on electric transmission ampacity and peak electricity load in the United States. *Environmental Research Letters*, 11.

Brown, M.A. et al., 2016. Modeling climate-driven changes in U.S. buildings energy demand. *Climatic Change*, 134(1–2), pp.29–44.

Byers, E.A. et al., 2016. Water and climate risks to power generation with carbon capture and storage. *Environmental Research Letters*, 11.

Byers, E.A., Hall, J.W. & Amezaga, J.M., 2014. Electricity generation and cooling water use: UK pathways to 2050. *Global Environmental Change-Human and Policy Dimensions*, 25, pp.16–30.

Carvalho, D. et al., 2017. Potential impacts of climate change on European wind energy resource under the CMIP5 future climate projections. *Renewable Energy*, 101, pp.29–40.

Chandramowli, S.N. & Felder, F.A., 2014. Impact of climate change on electricity systems and markets - A review of models and forecasts. *Sustainable Energy Technologies and Assessments*, 5, pp.62–74.

Chen, L., Pryor, S.C. & Li, D., 2012. Assessing the performance of intergovernmental panel on climate change AR5 climate models in simulating and projecting wind speeds over China. *Journal of Geophysical Research Atmospheres*, 117(24), pp.1–15.

Chum, H. et al., 2011. Bioenergy. *IPCC Special Report on Renewable Energy Sources and Climate Change Mitigation*.

De Cian, E., Lanzi, E. & Roson, R., 2013. Seasonal temperature variations and energy demand. *Climatic Change*, 116, pp.805–825.

Cradden, L.C. & Harrison, G.P., 2013. Adapting overhead lines to climate change: Are dynamic ratings the answer? *Energy Policy*, 63, pp.197–206.

Crook, J.A. et al., 2011. Climate change impacts on future photovoltaic and concentrated solar power energy output. *Energy & Environmental Science*, 4(9), p.3101.

Cruz, A.M. & Krausmann, E., 2013. Vulnerability of the oil and gas sector to climate change and extreme weather events. *Climatic Change*, 121(1), pp.41–53.

Deepthi, R. & Deo, M.C., 2010. Effect of climate change on design wind at the Indian offshore locations. *Ocean Engineering*, 37(11–12), pp.1061–1069.

Dowling, P., 2013. The impact of climate change on the European energy system. *Energy Policy*, 60, pp.406–417.

Durmayaz, A. & Sogut, O.S., 2006. Influence of cooling water temperature on the efficiency of a pressurized-water reactor nuclear-power plant. *International Journal of Energy Research*, 30(10), pp.799–810.

Ebinger, J. & Vergara, W., 2011. Climate Impacts on Energy Systems: Key Issues for Energy Sector Adaptation. *World Bank*.

Fant, C., Adam Schlosser, C. & Strzepek, K., 2016. The impact of climate change on wind and solar resources in southern Africa. *Applied Energy*, 161, pp.556–564.

FAO, 2016. The State of Food and Agriculture: Climate change, agriculture and food security. *Rome*.

Flörke, M., Teichert, E. & Bärlund, I., 2011. Future changes of freshwater needs in European power plants. *Management of Environmental Quality: An International Journal*, 22(1), pp.89–104.

Fuss, S. et al., 2014. Betting on negative emissions. *Nature Clim. Change*, 4(10), pp.850–853.

Gaudard, L. et al., 2014. Climate change impacts on hydropower in the Swiss and Italian Alps. *Science of the Total Environment*, 493, pp.1211–1221.

Gornall, J. et al., 2010. Implications of climate change for agricultural productivity in the early twenty-first century. *Philosophical Transactions of the Royal Society B*, pp.2973–2989.

Hamududu, B. & Killingtveit, A., 2012. Assessing climate change impacts on global hydropower. *Energies*, 5(2), pp.305–322.

Harrison, G.P. & Whittington, H.W., 2002. Vulnerability of hydropower projects to climate change. *IEE Proceedings-Generation, Transmission and Distribution*, 149(3), pp.249–255.

Hoffmann, B., Häfele, S. & Karl, U., 2013. Analysis of performance losses of thermal power plants in Germany - A System Dynamics model approach using data from regional climate modelling. *Energy*, 49(1), pp.193–203.

Hueging, H. et al., 2013. Regional changes in wind energy potential over Europe using regional climate model ensemble projections. *Journal of Applied Meteorology and Climatology*, 52(4), pp.903–917.

Ibrahim, S.M. a., Ibrahim, M.M. a. & Attia, S.I., 2014. The Impact of Climate Changes on the Thermal Performance of a Proposed Pressurized Water Reactor: Nuclear-Power Plant. *International Journal of Nuclear Energy*, 2014, pp.1–7.

Isaac, M. & van Vuuren, D.P., 2009. Modeling global residential sector energy demand for heating and air conditioning in the context of climate change. *Energy Policy*, 37(2), pp.507–521.

Kamranzad, B. et al., 2015. Climate change impact on wave energy in the Persian Gulf. *Ocean Dynamics*, 65(6), pp.777–794.

Karnauskas, K.B., Lundquist, J.K. & Zhang, L., 2017. Southward shift of the global wind energy resource under high carbon dioxide emissions. *Nature Geoscience*.

Khan, M.S.N. & Khan, M.M.A., 2014. Land Suitability Analysis for Sustainable Agricultural Land Use Planning in Bulandshahr District of Uttar. *International Journal of Scientific and Research Publications*, 4(3).

Koch, H. & Vögele, S., 2009. Dynamic modelling of water demand, water availability and adaptation strategies for power plants to global change. *Ecological Economics*, 68(7), pp.2031–2039.

Koch, H. & Vögele, S., 2013. Hydro-climatic conditions and thermoelectric electricity generation - Part I: Development of models. *Energy*, 63, pp.42–51.

Kopytko, N. & Perkins, J., 2011. Climate change, nuclear power, and the adaptation-mitigation dilemma. *Energy Policy*, 39(1), pp.318–333.

Kulkarni, S., Deo, M.C. & Ghosh, S., 2014. Changes in the design and operational wind due to climate change at the Indian offshore sites. *Marine Structures*, 37, pp.33–53.

Labriet, M. et al., 2015. Worldwide impacts of climate change on energy for heating and cooling. *Mitigation and Adaptation Strategies for Global Change*, 20(7), pp.1111–1136.

Langholtz, M. et al., 2014. Climate risk management for the U.S. cellulosic biofuels supply chain. *Climate Risk Management*, 3, pp.96–115.

Lesk, C., Rowhani, P. & Ramankutty, N., 2016. Influence of extreme weather disasters on global crop production. *Nature*, 529(7584), pp.84–87.

Linnerud, K., Mideksa, T.K. & Eskeland, G.S., 2011. The Impact of Climate Change on Nuclear Power Supply. *The Energy Jounral*, 32(1), pp.149–168.

Long, H. et al., 2013. Biomass resources and their bioenergy potential estimation: A review. *Renewable and Sustainable Energy Reviews*, 26, pp.344–352.

de Lucena, A.F.P. et al., 2009. The vulnerability of renewable energy to climate change in Brazil. *Energy Policy*, 37(3), pp.879–889.

de Lucena, A.F.P. et al., 2010. The vulnerability of wind power to climate change in Brazil. *Renewable Energy*, 35(5), pp.904–912.

Majone, B. et al., 2016. Impact of climate change and water use policies on hydropower potential in the south-eastern Alpine region. *The Science of the total environment*, 543(Pt B), pp.965–80.

Matko, M., Golobič, M. & Kontić, B., 2016. Integration of extreme weather event risk assessment into spatial planning of electric power infrastructure. *Urbani Izziv*, 27(1), pp.95–112.

McColl, L. et al., 2012. Assessing the potential impact of climate change on the UK’s electricity network. *Climatic Change*, 115(3–4), pp.821–835.

Mukheibir, P., 2013. Potential consequences of projected climate change impacts on hydroelectricity generation. *Climatic Change*, 121(1), pp.67–78.

Muller, C. & Robertson, R.D., 2014. Projecting future crop productivity for global economic modeling. *Agricultural Economics (United Kingdom)*, 45(1), pp.37–50.

Musilek, P., Arnold, D. & Lozowski, E., 2009. An ice accretion forecasting system (IAFS) for power transmission lines using numerical weather prediction. *Sola*, 5(December 2002), pp.2002–2005.

Olesen, J.E. et al., 2011. Impacts and adaptation of European crop production systems to climate change. *European Journal of Agronomy*, 34(2), pp.96–112.

Panagea, I.S. et al., 2014. Climate Change Impact on Photovoltaic Energy Output: The Case of Greece. *Advances in Meteorology*, 2014, pp.1–11.

Parkinson, S. & Djilali, N., 2015. Robust response to hydro-climatic change in electricity generation planning. *Climatic Change*, pp.475–489.

Pašičko, R., Branković, Č. & Šimić, Z., 2012. Assessment of climate change impacts on energy generation from renewable sources in Croatia. *Renewable Energy*, 46, pp.224–231.

Patt, A., Pfenninger, S. & Lilliestam, J., 2013. Vulnerability of solar energy infrastructure and output to climate change. *Climatic Change*, 121, pp.93–102.

Porter, J.R. et al., 2014. Food security and food production systems. *Climate Change 2014: Impacts, Adaptation, and Vulnerability. Part A: Global and Sectoral Aspects. Contribution of Working Group II to the Fifth Assessment Report of the Intergovernmental Panel on Climate Change*.

Pryor, S.C. & Barthelmie, R.J., 2013. Assessing the vulnerability of wind energy to climate change and extreme events. *Climatic Change*, 121(1), pp.79–91.

Pryor, S.C. & Barthelmie, R.J., 2010. Climate change impacts on wind energy: A review. *Renewable and Sustainable Energy Reviews*, 14(1), pp.430–437.

Reed, D.A., 2008. Electric utility distribution analysis for extreme winds. *Journal of Wind Engineering and Industrial Aerodynamics*, 96, pp.123–140.

Reeve, D.E. et al., 2011. An investigation of the impacts of climate change on wave energy generation: The Wave Hub, Cornwall, UK. *Renewable Energy*, 36(9), pp.2404–2413.

Rosenzweig, C. et al., 2014. Assessing agricultural risks of climate change in the 21st century in a global gridded crop model intercomparison. *Proceedings of the National Academy of Sciences of the United States of America*, 111(9), pp.3268–73.

Rubbelke, D. & Vögele, S., 2011. Impacts of climate change on European critical infrastructures: The case of the power sector. *Environmental Science and Policy*, 14(1), pp.53–63.

Santos, J.A. et al., 2015. Projected changes in wind energy potentials over Iberia. *Renewable Energy*, 75(2015), pp.68–80.

Schaeffer, R. et al., 2012. Energy Sector Vulnerability to Climate Change: A Review. *Energy*, 38(1), pp.1–12.

Schauberger, B. et al., 2017. Consistent negative response of US crops to high temperatures in observations and crop models. *Nature Communications*.

Seljom, P. et al., 2011. Modelling the effects of climate change on the energy system-A case study of Norway. *Energy Policy*, 39(11), pp.7310–7321.

Sieber, J., 2013. Impacts of, and adaptation options to, extreme weather events and climate change concerning thermal power plants. *Climatic Change*, 121(1), pp.55–66.

Spalding-Fecher, R. et al., 2016. The vulnerability of hydropower production in the Zambezi River Basin to the impacts of climate change and irrigation development. *Mitigation and Adaptation Strategies for Global Change*, 21(5), pp.721–742.

Stromberg, P.M., Esteban, M. & Gasparatos, A., 2011. Climate change effects on mitigation measures: The case of extreme wind events and Philippines’ biofuel plan. *Environmental Science and Policy*, 14(8), pp.1079–1090.

Tobin, I. et al., 2015. Assessing climate change impacts on European wind energy from ENSEMBLES high-resolution climate projections. *Climatic Change*, 128, pp.99–112.

Tuck, G. et al., 2006. The potential distribution of bioenergy crops in Europe under present and future climate. *Biomass and Bioenergy*, 30(3), pp.183–197.

Turner, S.W.D., Yi, J. & Galelli, S., 2017. Examining global electricity supply vulnerability to climate change using a high-fidelity hydropower dam model. *Science of the Total Environment*, 591, pp.663–675.

Tyusov, G.A. et al., 2017. Projected climate change impacts on the operation of power engineering facilities in Russia. *Russian Meteorology and Hydrology*, 42(12), pp.775–782.

Vicuña, S., Dracup, J. a. & Dale, L., 2011. Climate change impacts on two high-elevation hydropower systems in California. *Climatic Change*, 109(S1), pp.151–169.

van Vliet, M.T.H. et al., 2016. Power-generation system vulnerability and adaptation to changes in climate and water resources. *Nature Climate Change*, (January).

van Vliet, M.T.H., Vögele, S. & Rübbelke, D., 2013. Water constraints on European power supply under climate change: impacts on electricity prices. *Environmental Research Letters*, 8, p.35010.

Wang, W. et al., 2014. Predicting the impacts of climate change on the potential distribution of major native non-food bioenergy plants in China. *PLoS ONE*, 9(11), pp.1–11.

Wilbanks, T. et al., 2014. Climate Change and Energy Supply and Use: Technical Report for the U.S. Department of Energy in Support of the National Climate Assessment.

Winkler, J. et al., 2010. Performance assessment of topologically diverse power systems subjected to hurricane events. *Reliability Engineering and System Safety*, 95(4), pp.323–336.

Zabel, F., Putzenlechner, B. & Mauser, W., 2014. Global agricultural land resources - A high resolution suitability evaluation and its perspectives until 2100 under climate change conditions. *PLoS ONE*, 9(9).
